# From avatars to body swapping: The use of virtual reality for assessing and treating body‐size distortion in individuals with anorexia

**DOI:** 10.1002/jclp.22724

**Published:** 2018-12-15

**Authors:** Silvia Serino, Nicoletta Polli, Giuseppe Riva

**Affiliations:** ^1^ Department of Psychology Università Cattolica del Sacro Cuore Milan Italy; ^2^ Istituto Auxologico Italiano IRCCS Applied Technology for Neuro‐Psychology Lab Milan Italy; ^3^ Istituto Auxologico Italiano, IRCCS Division of Endocrinology and Metabolism Milan Italy; ^4^ Department of Clinical Sciences and Community Health University of Milan Milan Italy

**Keywords:** anorexia nervosa, avatar, body‐size distortion, full body illusion, virtual reality

## Abstract

In the last 30 years, virtual reality (VR) has offered innovative solutions for assessing and treating body representation disturbances in anorexia nervosa (AN). The most recent and innovative trend is the exploitation of the so‐called VR‐based body swapping illusion. The aim of this case study was to report the use of this VR protocol within a multidisciplinary treatment of AN. A patient with a Diagnostic and Statistical Manual‐5 diagnosis of AN underwent an intensive multidisciplinary outpatient treatment. Three sessions of a VR‐based body swapping illusion (i.e., the experimental induction of being the owner of a virtual body as a result of a visuotactile stimulation) were delivered within the treatment protocol (i.e., beginning of the treatment; end of one cycle of the treatment; 1 year of follow‐up). We report the results obtained, discussing how the VR full body illusion was both able to effectively monitor changes of multisensory bodily integration and to act as a driver for these changes.

## 1 INTRODUCTION

The prevalence of anorexia nervosa (AN) has been reported to be about 1% in women and less than 0.5% in men; in the past several years, an increased risk for adolescents (i.e., 15–19‐year old girls) has been noted (Smink, Van Hoeken, & Hoek, 2012; Zipfel, Giel, Bulik, Hay, & Schmidt, 2015). Despite the substantial advances in assessing and treating AN, this serious eating disorder still represents an unresolved problem (Zipfel et al., 2015).

Two recent longitudinal studies examined the influence of such factors as body dissatisfaction, self‐objectification, appearance‐ideal internalization, dieting, and negative affectivity in predicting both Diagnostic and Statistical Manual‐5 (DSM‐5; American Psychiatric Association, 2013) eating disorders onset and maintenance at 4‐year follow‐up. The two studies, involving more than 5,000 male and female college students (Dakanalis, Clerici, et al., 2016; Dakanalis et al., 2017), underlined the critical role played by the experience of the body, and in particular self‐objectification (thinking about and monitoring the body’s outward appearance from a third‐person perspective), for the initiation and the persistence of all eating disorders. Interestingly, this variable explained between two and three times more the emergence and maintenance of the disturbances than both dieting and negative affectivity. According to a recent neuroscientific framework (Dakanalis, Gaudio, et al., 2016; Riva, 2014, 2018; Riva & Dakanalis, 2018; Riva & Gaudio, 2018), this altered experience of the body may reflect a deficit in the processing and integration of multisensory bodily representations that are shaped by top‐down predictive mechanisms (Riva, 2018; Riva & Dakanalis, 2018).

A deficit of these processes may impair the possibility in individuals with AN of revising their representation of the body (third‐person, offline) with new content from real‐time perception‐driven inputs (first‐person, online), locking them into an old and negative memory of the body that cannot be changed even after a demanding diet or significant weight loss.

In the last 30 years, virtual reality (VR) has offered innovative solutions for assessing and treating body representation disturbances in AN (Ferrer‐García & Gutiérrez‐Maldonado, 2012, 2013). The possibility of developing VR‐based applications specifically targeting body representation disturbances has advanced due to substantial progress in technology that now support the use of increasingly realistic and interactive “avatars.” The term “avatar” refers to the virtual self‐representations in digital worlds, including massively multiplayer online role‐playing games (e.g., World of Warcraft), online collaborative virtual worlds (e.g., second life), and videogames and virtual environments for clinical purposes (Gaggioli, Mantovani, Castelnuovo, Wiederhold, & Riva, 2003).

The most recent and innovative trend for treating body representation disturbances in AN is the exploitation of the so‐called VR‐based body swapping illusion (Serino & Dakanalis, 2017; Serino et al., 2016; Slater, Spanlang, Sanchez‐Vives, & Blanke, 2010). Advances in synchronous multisensory stimulation technology permit the experimental induction of viewing an entire virtual body from a first‐person perspective, so it is perceived as one’s own real body.

Virtual environments allow the development of complex scenarios that are very close to those in the daily life. Moreover, these synthetic settings allow complete control over the stimuli delivered and the virtual scenarios can be considered “safe places” where patients can confront feared situations without risks.

Riva, Bacchetta, Baruffi, Rinaldi, and Molinari (1999) provided a pioneering example with the Body Image Virtual Reality Scale (BIVRS), a specific VR‐based application for assessing body‐size distortions in eating disorders. Participants select from among nine three‐dimensional (3D) figures that range from being significantly underweight to being significantly overweight to indicate how they perceived themselves. The BIVRS was also used in the development of the virtual environment for body image modification (Riva, Bacchetta, Baruffi, Rinaldi, & Molinari, 1998), one of the first applications of VR in the context of eating disorders treatment. Specifically, patients were exposed to photographs of their real bodies that had been digitalized. The protocol included three phases. Users first were exposed to digitalized photographs of underweight, normal weight, and overweight people. They then discussed their feelings that emerged from these images. Finally, they had the option to model their perceived body image using a 3D figure to compare their actual and ideal body representations.

Uses of VR have continued to develop since these early innovative proposals. In fact, two recent systematic reviews concluded that VR offers significant advantages for the assessment and treatment of body representation disturbances (Ferrer‐García & Gutiérrez‐Maldonado, 2012; Ferrer‐Garcia et al., 2013). One of those advantages is the possibility to interact with realistic avatars that represent both the perceived and ideal body representation. Moreover, the use of immersive VR‐based applications made possible by head‐mounted displays allows participants to experiment with a high level of “presence” (the feeling of “being” in the virtual scenarios). Patients can be confronted with their own virtual body in the virtual environment.

More recently, increased efforts to understand the multisensory nature of body representation disturbances in AN, combined with the rapid development of immersive VR technology, have led to the emergence of new protocols, including the VR‐based body swapping illusion. The illusory ownership over a virtual body is achieved by observing a synchronous stimulation from a first‐person perspective. Specifically, participants perceive a stimulation on their actual body while they see the exact same stimulation on the virtual body rendered in the VR headset. The usual control condition is an asynchronous stimulation. That is, participants perceive a stimulation on their actual body that has a different timing from the what they see through the VR headset.

Keizer, Smeets, Postma, van Elburg, and Dijkerman (2014) and Keizer, van Elburg, Helms, and Dijkerman (2016) were the first to find that VR‐based body swapping was able to decrease body‐size overestimation in patients suffering from AN. These findings are consistent with a growing body of studies indicating that embodiment in an artificial body with different dimensions alters body representations to be more congruent with the embodied avatar. For example, Preston and Ehrsson (2014) used a thin avatar to reduce overestimation in a sample of female participants.

Taken together, these studies suggest a clinical application for methods that specifically target multisensory body integration deficits (for a review, see Serino & Dakanalis, 2017). Nonetheless, there are no available instruments for evaluating and treating multisensory body integration deficits in clinical settings.

The aim of this case study was to report the use of VR‐based body swapping within a multidisciplinary treatment of AN. A patient with a DSM‐5 diagnosis of AN underwent an intensive outpatient treatment of this disorder within a multidisciplinary model that included different therapeutic activities such as art therapy, group therapy, and psychoeducational interventions. Three sessions of a VR‐based body swapping illusion (the experimental illusion of being the owner of a virtual body thanks to a visuotactile stimulation) were delivered: one at the beginning of the treatment (Time 1), one at the end of a cycle of the treatment (Time 2), and one at 1‐year follow‐up (Time 3). We report here the results obtained over these three times by asking the patient to estimate her body before the illusion and after each condition (i.e., synchronous vs. asynchronous).

### 1.1 Presenting problem and client description

Andrea (a pseudonym) was in her 30s when she was admitted to the Eating Disorders Centre, Division of Endocrine and Metabolic Diseases, San Luca Hospital in Milan, Italy. She is a child of a couple who are in the process of legally separating but currently living together. Her first eating‐related disorder began to emerge in late puberty. Following several difficulties connected to the family environment, Andrea started a restrictive diet, losing 13 kg (28.6 lbs) in the span of a year (body mass index [BMI] = 14.8). Acknowledging her distress, she started group therapy, regaining the lost weight (BMI = 22.4) after 2 years. In the following years, Andrea manifested restrictive eating tendencies that affected her weight slightly.

Between ages 23 and 25, Andrea reported a period of healthy behaviors and social proactivity. She started positive relationships, employment, and long‐term life projects. This period of relative peace and functionality lead to Andrea’s major decision to purchase an apartment to live in on her own. Her feelings regarding this new home were complex and she reported that she never fully experienced the new home as her own. In fact, she deeply desired to relocate to another city. In this period, Andrea restarted a series of eating restriction behaviors. She lost weight in a very short period, reaching 31.7 kg (70 lbs; BMI = 12.5) when she was 27‐years old.

In the same year, she voluntary entered the eating disorder ward of Hospital Niguarda (Milan, Lombardy, Italy), starting a focused nutritional therapy (i.e., assisting patients in reaching a balanced relationship with food to meet daily nutritional needs) that helped her regain a barely functional weight (BMI = 18.5). When finished the first cycle of the treatment (after 3 months), Andrea started individual psychotherapy along with group therapy. Both were still in progress at the time of this report. Nonetheless, following the release from the Hospital Niguarda, Andrea started to progressively restrict her caloric intake, consequently losing weight.

In 2016, Andrea finally requested admission to our center for rehabilitative support. She presented herself to our unit with a weight of 32 kg (70.5 lbs; BMI = 13.69), severe distortions in body perception, and several specific idiosyncrasies connected to the food (i.e., she was not able to cook and eat because she was not able to tolerate the smell of food). Andrea was unemployed as she was unable to sustain the physical and psychological effort required of continuous work.

### 1.2 Case formulation

Andrea met all the DSM‐5 criteria for a diagnosis of AN, restrictive subtype. Moreover, she presented highly pervasive disturbances in body perception that suggested she was a fitting candidate for the VR‐based body swapping protocol.

Because of the chronicity of her clinical condition, Andrea’s metabolic and endocrine levels were severely compromised. Mineralogical panels also indicate depletion of bone tissues, with severe osteoporosis. Nonetheless, in contrast to past therapeutic experiences, Andrea’s insight regarding her physical condition as well as her motivation to engage in this treatment seemed strong.

### 1.3 Course of treatment

The program entails a multidisciplinary approach that includes several experts (endocrinologists, psychiatrists, psychologists, and dietitians) who collaborate in an outpatient rehabilitative service. The program lasts for 12 weeks, with session frequency determined by the specific clinical, physiological, and psychological needs of each patient.

During the treatment, Andrea made significant changes in her eating patterns, at both a quantitative and qualitative level. Improvements in both her psychological and physical functioning allowed Andrea to resume work, one of her stated goals. She maintained good insight regarding her condition and a steady desire to improve. After a long observation period, her psychiatrist decided to start Andrea on an antidepressant medication (sertraline) with subsequent improvement of her general mood and obsessive food‐related behaviors. Andrea also was introduced to a novel clinical and therapeutic instrument that utilizes VR full body illusion to evaluate changes in multisensory bodily integration.

We administered the VR‐based protocol at the beginning (Week 1) and end (Week 12) of the therapeutic cycle, and then again at 1‐year follow‐up. The hospital unit then recommended that Andrea engages in another cycle of intervention. Nonetheless, she decided to leave the Outpatient Rehabilitative Service. The decision was motivated by her insights regarding the importance of health‐related choices in her life; this new awareness was reflected in a healthier weight (BMI = 17.66). She started new job work, a new social life, and demonstrated general improvement in her relationships with her family.

## 2 MEASURES

Two types of measures were used to evaluate Andrea’s response to VR full body illusion: one assessing the accuracy of her body perceptions (i.e., *Body‐size estimation task*), the other assessing the level of her embodiment of the avatar (i.e., the *Embodiment Questionnaire*). These measures were administered after each (synchronous/asynchronous) condition across the three sessions (i.e., beginning of the treatment; end of one cycle of the treatment; 1‐year follow‐up).

### 2.1 Body‐size estimation task

To monitor changes in multisensory bodily integration we asked Andrea to perform a “body‐size estimation task” (see Keizer et al., 2014 and Serino et al., 2016). She was requested to provide an estimate of three different parts of her body (i.e., shoulders, abdomen, and hips) under two different conditions. This procedure was consistent with previous studies in the embodiment field that have indicated that the illusion works specifically on the upper part of the body (Keizer et al., 2014; Serino et al., 2016). In the “*body‐size estimation task—width*,” she was asked to use two adhesive stickers and place them on a white wall to indicate the estimated distance between the abovementioned body parts (e.g., the estimated distance between the right and the left hip). Because it was important that these estimates reflect her own inferences and memories of her body, she was asked to not look at her body while engaged in this task. Then, in the “*body‐size estimation task—circumference*,” she was asked to estimate the circumference of the same body parts with a piece of small cotton rope, placed on a table.

She performed the entire self‐measuring procedure three times: before the induction of the illusion (“baseline”) and after each of the two conditions of the illusion (i.e., “postsynchronous stimulation” and “postasynchronous stimulation”). In short, three times for each of the three VR sessions, for a total of nine measures. At the end of each session, the *actual* measures Andrea’s hips, abdomen, and shoulders (width and circumference) were collected to evaluate the congruence between Andrea’s estimations and her real bodily dimensions, that is an “index of misestimation” (Keizer et al., 2014).

### 2.2 Embodiment Questionnaire

To evaluate her sense of being in the virtual body, Andrea was asked to complete a 15‐item Embodiment Questionnaire (Piryankova et al., 2014; Serino et al., 2016) after the “synchronous visuotactile stimulation” (experimental condition) and the “asynchronous visuotactile stimulation” (control condition). The questionnaire's scales evaluate three dimensions of embodiment: *ownership* (i.e., the sense of being the owner of the virtual body; 11 items), *self‐location* (i.e., the sense of being in the same spatial location of the virtual body, 3 items), and *agency* (i.e., the sense of being the agent of movement of the virtual body, 3 items). The three subscales were calculated as the mean of items. This questionnaire was used previously in similar studies (Keizer et al., 2014; Serino et al., 2016).

### 2.3 VR‐based body swapping

**Figure 1:**
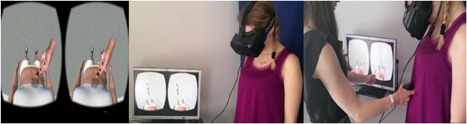
Virtual reality (VR) full body illusion [Color figure can be viewed at wileyonlinelibrary.com]

The VR‐based body swapping illusion is the experimental induction via multisensory stimulation of being the owner of a virtual body. To achieve this illusion, we used a virtual body of a young and thin healthy‐weight woman (i.e., a virtual body of different size in comparison of actual body of the patient) standing upright in a stimulus‐free room (Figure 1). The waist circumference of the avatar was 73.95 cm. She wore a white top and shorts. The avatar was modeled with the software MakeHuman (www.makehuman.org), while the virtual room was developed with the software Unity3D (www.unity3d.com). To visualize the virtual body, Andrea was asked to wear a head‐mounted display (HMD, Oculus Rift DK2) connected to a portable computer. A Razer Hydra motion‐tracking device connected to the portable computer was used to induce the multisensory stimulation.

The illusion requires two conditions, namely the “synchronous visuotactile stimulation” (experimental condition) and the “asynchronous visuotactile stimulation” (control condition). In the “synchronous visuotactile stimulation,” Andrea visualized the avatar in first‐person perspective and was asked to lift her shirt to expose her abdomen. Then, the clinician provided a tactile stimulation on her abdomen for about 90 s with a brush connected to the motion‐tracking device. At the same time, Andrea had the opportunity to visualize a virtual brush touching the abdomen of the virtual avatar (Figure 1) in synchronicity to the clinician's movements.

Consequently, there was a perfect synchrony between what she perceived on her actual body (i.e., tactile stimulation) and what she saw (i.e., visual inputs) on the avatar’s body. This has been demonstrated to elicit ownership over the virtual body (Serino & Dakanalis, 2017; Serino et al., 2016).

In the “asynchronous visuotactile stimulation” Andrea visualized the avatar in first‐person perspective and was invited to lift her shirt as well. However, the visuotactile stimulation was somewhat different. The clinician provided an asynchronous tactile stimulation on her abdomen with the brush for about 90 s. Specifically, there was a delay between what she perceived on her actual body (i.e., tactile stimulation) and what she saw (i.e., a delayed visual input) on the virtual body. Andrea therefore perceived an incongruent situation where her body and the virtual avatar received mismatching inputs. This condition has been demonstrated to not elicit ownership over the virtual body, and therefore it is used as control condition (Serino & Dakanalis, 2017; Serino et al., 2016).

### 2.4 Procedure

At the start of the session, Andrea was provided with information about the objectives and the structure of the protocol. Specifically, she was informed that this VR‐based protocol was aimed at monitoring changes in her perception of the body during the progression of her treatment with advanced immersive technology. This introduction helped Andrea to understand the purposes of this VR‐based session and to become familiar with the setting.

Then, she was invited to complete the Body‐size estimation task by estimating the width and the circumference of her shoulders, abdomen, and hips. Subsequently, she was asked to wear the HMD‐Oculus Rift DK2 to experience the VR body‐swap illusion. As explained, the VR body illusion consisted of two different conditions: “synchronous visuotactile stimulation” and “asynchronous visuotactile stimulation.” Each condition lasted 90 s, where Andrea saw the virtual avatar and experienced the tactile stimulation provided by the clinician on her abdomen. According to the condition (treatment vs. control), this real touch could be synchronous to the virtual touch on the virtual avatar or not. After each virtual experience, there was a brief break (5 min), and then Andrea was asked to perform the Body‐size estimation task and complete the Embodiment Questionnaire.

## 3 RESULTS

### 3.1 Embodiment Questionnaire

At the beginning of the treatment (Time 1), results from the Embodiment Questionnaire indicated that the illusion was quite successfully induced in both synchronous and asynchronous conditions as scores were high on all three dimensions (i.e., ownership, self‐location, and agency; Table 1).

**Table 1:** Embodiment Questionnaire results by condition (postsynchronous stimulation vs. postasynchronous stimulation) × time (treatment beginning, treatment ending, and 1‐year posttreatment)

	Time 1	Time 2	Time 3
Ownership			
Synchronous condition	2.56	1.78	1.68
Asynchronous condition	4.33	1.25	3.75
Self‐location			
Synchronous condition	2.25	1	2
Asynchronous condition	1.75	1	1
Agency			
Synchronous condition	2	1	1.5
Asynchronous condition	3	1	1

*Note*. Possible scores ranged from 1 to 5, where higher scores meant higher feelings of embodiment of avatar.

Nonetheless, Andrea’s verbal expressions during the VR protocol indicated that she was more embodied in the synchronous conditions. As per the English translation of her report: “I felt the virtual body as it was my own; when I was looking at my body from top to bottom, it was exactly as the virtual one and this allowed me to identify myself with it.” And in contrast: “I saw the body too high, I did not identify myself with it very much.”

A different pattern of results emerged at the end of treatment (Time 2), and after 1 year of follow‐up (Time 3). Specifically, the patient reported lower scores in both conditions, indicating that she did not experience the illusion in terms of body ownership, self‐location, and agency. It is noteworthy that she showed high scores on the ownership subscale in the “asynchronous visuotactile condition” at Time 3, which was reflected in the changes in bodily experience measured with the body‐size estimation task. It is therefore possible to conclude that the illusion was successfully reached only at Time 1, as also evidenced by the effect on body perception discussed in the following section.

### 3.2 Body‐size estimation task—Width

At the beginning of the treatment, Andrea exhibited an overestimation of all body parts for which she was asked to make estimates. The embodiment in virtual body in the “synchronous visuotactile condition” was partially able to reduce this overestimation, but Andrea persisted in these distortions after the induction of the illusion. As an example, it is possible to consider the findings obtained for the estimation of width of the hips. At baseline, she manifested a dramatic overestimation in the dimension of her hips (94.44)—that is, estimating her hips at nearly twice their actual size. After the “synchronous visuotactile condition,” there was a significant decrease in the percentage of misestimation (38.89%), which was not reflected in the estimates collected after the asynchronous condition (77.78%).

A completely different pattern of results was obtained at the end of the treatment (Time 2). At this point, Andrea overestimated all her relevant body parts, but in contrast to Time 1, the illusion did not succeed in reducing these distortions, as reflected also in low scores obtained from the Embodiment Questionnaire.

At Time 3, Andrea’s estimates of body size were quite accurate. Accordingly, she returned to the follow‐up with a markedly improved status, along with several significant‐positive changes in her life. This general improvement in her wellbeing was reflected in her weight and in the reduction of her body representation disturbances with VR body protocol acting as “multisensory driver” for these positive changes in body perception. After the embodiment in the virtual body in both synchronous and asynchronous condition, Andrea showed a tendency to correctly estimate the width of her body. In regard to the estimation of her hips. Andrea had an abnormal and quite “negative” reaction to the “asynchronous visuotactile condition” (i.e., the control condition). In that instance, she manifested a dramatic increase in overestimation. These results coherently aligned with the scores on the Embodied Questionnaire reported in the previous paragraph.

### 3.3 Body‐size estimation task—Circumference

We observed a similar pattern of results in regard to circumference as what we collected for width estimations, especially for Time 1 and Time 2.

At the beginning of the treatment, Andrea exhibited a large overestimation of her body size. The VR‐based body swapping illusion—especially after the “synchronous visuotactile condition”—appeared to partially and temporarily reduce these dramatic distortions. For example, the overestimation manifested in the perception of the abdomen (46.55%) was reduced after the synchronous stimulation (19.83%). Conversely, the effect was only weakly present in the asynchronous condition (30.17%). At Time 2, at the end of the treatment, she manifested some distortions in the perception of her body, although she was quite accurate in the estimations of the shoulders; probably because abdomen and hips are more emotionally‐laden sites. After the embodiment in the virtual body in both conditions, there were no appreciable differences in her estimates; the scores on the Embodiment Questionnaire indicated, indeed, that the illusion did not work in these sessions.

At 1‐year follow‐up (Time 3), Andrea showed a large underestimation of her shoulders. After the induction of the illusion with a synchronous visuotactile stimulation, she demonstrated marked reduction of the body distortion. She perceived her body with more accuracy and objectivity. Nonetheless, she exhibited an overestimation of the circumference of the abdomen, which significantly decreased after the illusion in both conditions.

Her perception of her hips was initially quite accurate. But here too, it was possible to observe an abnormal and quite negative reaction to the asynchronous visuotactile condition: Andrea showed a large overestimation after this manipulation.

## 4 SUMMARY

This clinical case highlights the potential application of “virtual embodiment” as an innovative instrument that is able both to effectively monitor changes of multisensory bodily integration both to act as a driver for these changes. As described in literature, individuals with AN demonstrated profound alterations in multisensory integration, which represents a fundamental risk factor as well as a core maintenance element of this pathology. The relevance of multisensory integration in AN is essential, nonetheless the field has been limited by the few instruments available to assess and treat these specific integration processes. Andrea’s case demonstrates how a VR‐based body swapping illusion can work as a useful and powerful instrument to assess and to modify distortions in multisensory integration. Furthermore, results indicated that this assessment tool can also promote posttreatment effects. As expected and in keeping with previous literature (Serino & Dakanalis, 2017) the VR‐based assessment protocol demonstrated clinical and therapeutic possibilities: the illusion was able to modify Andrea’s body experience. This also was indicated by Embodiment Questionnaire scores indicating that the illusion was successfully induced.

At her admission in the hospital ward, Andrea presented with severe and pervasive distortions in her body image. Following the VR‐based protocol both the clinical observations and the data collected indicated partial but significant reduction of these distortions. At Time 1, we found that the illusion successfully worked and the patient showed an improvement in the estimation of her body. Therefore, we can suppose that the protocol can work as a “multisensory driver” for the cognitive restructuring processes, which is at the heart of this multidisciplinary treatment. These results support previous studies that showed how avatars can alter bodily experiences in clinical and healthy subjects, inducing temporary but pervasive changes in the perceived body following the direction of the illusions (i.e., embodiment in a smaller body can reduce the estimated size of the real body; Serino et al., 2016).

However, to fully exploit its potentiality as a treatment instrument, it will be necessary to further test the efficacy of VR body swapping using repeated sessions within an integrated therapeutic approach for body‐size disturbances. More, what makes our body so special is that, unlike other physical objects, we also have an internal access to it through inner (interoceptive, proprioceptive and vestibular) signals. So, simulative technology should also modulate the internal/inner body experience to allow the development of a complete synthetic multisensory experience (virtual embodiment). In particular, an emerging technology – Sonoception, the use of wearable acoustic and vibrotactile transducers to alter internal bodily signals – may be the missing piece: it was able to enhance heart rate variability (the short‐term vagally mediated component—rMSSD) through the modulation of the subjects' parasympathetic system (Di Lernia, Cipresso, Pedroli, & Riva, 2018). As part of a therapeutic strategy, the “virtual embodiment” in an avatar may represent an innovative method to specifically reduce bodily concerns. It is worthy to note that the exploitation of VR also in clinical practice has been limited for many years because of the high cost of virtual equipment and the difficulty in set‐up virtual environments without sufficient programming skills. Today, VR technology field is changing dramatically, thanks to broad availability of low‐cost and user‐friendly VR platforms (Oculus Rift^©^ and HTC Vive™ (Castelvecchi, 2016, Riva, Serino, Di Lernia, Pavone, & Dakanalis, 2017), which permit to have virtual experiences at a very reasonable price—less than $3,000 for a fully configured system. Moreover, even more affordable solutions are available for clinical practice, based on smartphones and tablets. With a low‐cost head‐mounted display connected to a smartphone, user are completely immersed in a virtual environment by controlling the viewing direction thanks to head movements, as in other VR set‐up. In this scenario, it will be possible to easily set‐up tailored VR sessions with avatars presented in different perspectives (third vs. first‐person), targeting in a powerful way the complex nature of body representations disturbances in AN with a powerful yet accessible new instrument (Riva, 2018).
